# If prognosis allows

**DOI:** 10.1017/S1478951525101570

**Published:** 2026-01-08

**Authors:** Henry Bair

**Affiliations:** Wills Eye Hospital, Philadelphia, PA, USA

I used to think my role in seeing ICU patients was straightforward: check the eyes, write my note, and move out of the way of those keeping the patient alive. On a particular Tuesday night, I saw how even a “simple” ophthalmology consult can be part of how we care for patients at the end of life. I was the ophthalmology resident on call when my phone vibrated with a new consult request.

It came from the MICU: “cloudy eye,” ventilated patient, rule out ulcer.

She was seventy-two, according to the chart. Stage IV lung cancer. ARDS. Septic shock. Pressors, dialysis, tube feeds, a list of drips that amounted to *everything, all at once, all the time*.

Her name was Mrs. L. I confirmed it from the bracelet on her wrist, not from her lips, which were parted only enough for the endotracheal tube. The ventilator sighed and pushed and sighed again.

“Her right eye looks hazy now,” the nurse said, keeping her voice low, as if the patient might be sleeping instead of sedated and paralyzed. “Family was worried. Attending wanted ophtho to take a look.”

We are very good at taking a look.

I barely needed to open her eye. The lid came up halfway on its own: lagophthalmos, exposure. The cornea was dull and stippled, the once-clear surface now with a faint grayness.

I turned on my penlight and watched the beam sweep across the eye. There it was: an irregular opacity near the bottom, right where the cornea had been drying out. Early ulcer. One more complication in a body already crowded with them.

I did what I had been trained to do.

Fluorescein strip. Cobalt blue light. A vague crescent of uptake. No hypopyon. Pupil sluggish but symmetric. I documented my findings in the workstation next to the bed. Assessment and plan: exposure keratopathy with early ulceration in the setting of prolonged intubation and incomplete lid closure. Recommend lubricating ointment every 2 hours, moisture chamber, gentle taping of the lids between checks, consider temporary tarsorrhaphy if prognosis allows.

*If prognosis allows.*

Her daughter had slipped into the room, wrapped in a cardigan too thin for the air-conditioned chill. She watched me with the particular intensity of someone who hasn’t understood most of what has happened for days but has learned to track who is new.

“You’re the eye doctor?” she asked.

“Yes.”

“Is she…going blind now too?”

I thought about all the things I had read in her chart. Fluid overload. Kidney failure. Congestion hepatitis. Respiratory failure. Encephalopathy. I had read the phrase “this is likely a terminal hospitalization” in a recent attending physician note. There was part of me that wanted to blurt out *this ulcer is the least of her problems.*

“Her eye is dry and a little infected,” I managed. “That’s usually uncomfortable. We can’t ask her, but I’m going to start some treatments to protect the surface of the eye, so if she wakes up, she’s more likely to see comfortably.”

Her daughter nodded slowly. “She loves to read,” she said. “Or she used to. She always said if she lost her eyesight, that would be the end.”

I looked at the tangled wires and lines hooked up to her body, thought of the “Full code” status in the chart. I wondered what “the end” meant here, and for whom.

The palliative care team often talks about goals of care, values, priorities. They schedule family meetings, gather chairs, turn their phones to silent for a set block of time. They say “big picture” and “where we go from here.”

Ophthalmology is rarely in those meetings. We stop by instead when everyone else is gone, with fluorescein strips and ointment, asked to protect a clear cornea while so much behind the eye – and everywhere else – is falling apart.

Later, at a nursing station nearby, I finalized my note. The cursor blinked after *if prognosis allows*. I left the phrase, even though I had a sense of the prognosis. The nurses knew. The family probably knew, somewhere underneath hope and fear. The only person who didn’t appear to know, at least according to my note, was me.

Days later, I checked the chart. Mrs. L had died early Friday morning. Time of death: 05:16. Of course, there was no mention of the corneal ulcer anywhere in the final note.

The orders I’d written for lubricating ointment were still there, now labeled “discontinued.” Scrolling through the medication list, I was struck by how few of our active orders, mine included, had been written primarily for her comfort. The eye ointment might have been one of them. Almost everything else had been in service of keeping her alive as long as possible.

In the weeks afterward, I kept thinking about that consult – not just about the ulcer, but about how little attention we give to vision when a patient is clearly dying. We manage blood pressure, oxygenation, pain, delirium, but rarely ask what the world actually looks like to the person in the bed, or whether they can still see the people gathered around them.

There is a particular cruelty in losing sight near the end of life. Much of how we say goodbye is visual: the last look at a loved one’s face, the familiar disorder of a home, the light through a window. We may talk about “hearing” each other, but we make sense of decline with what we see. “It doesn’t look good.” “We saw it coming.” “I couldn’t bear to see her like that.”

Palliative care has long been comfortable with pain, nausea, anxiety. Eyes often show up as an afterthought – artificial tears added to an order set, “lubricate as needed” typed in by whoever remembers. Yet I have seen patients in their last days become more distressed by dry, burning eyes, harsh overhead lights, or dirty glasses than by abstract fears of death.

These are small problems. But in the final stretch, small problems are sometimes what people actually feel.

So I find myself wondering: what would it mean to treat eye comfort as a core palliative symptom rather than cosmetic maintenance? To ask, as routinely as we ask about pain, whether patients can see the faces around them, whether the light bothers them, whether their eyes feel dry, gritty, or burning? To invite ophthalmology into conversations not only about whether we can save vision, but also about when it is kinder to stop chasing small gains and focus on comfort instead?

I know lubricating ointment does not change the course of metastatic cancer. A moisture chamber will not reverse multi-organ failure. But I also know what it feels like to lie awake with dry, burning eyes after a long day, contacts left in too long, the pain out of proportion to the size of the organ involved. If that small discomfort can dominate the night of a young, healthy resident, what might it mean to someone whose body is already under siege?

Sometimes, late on call, I think of Mrs. L and her daughter’s question: would her mother be able to see them if she woke up? There are questions medicine cannot answer, no matter how many consults we place. But there are questions we can at least honor – by dimming the light, by closing an eyelid, by softening how the world reaches a person whose world is shrinking.

In the ICU, in the ER, in the quiet exam lanes of clinic, I am learning that palliative care does not begin at a particular lab value or at the moment someone signs hospice papers. Sometimes, it begins with noticing a cloudy eye in a room full of louder alarms, and choosing to treat that eye – not as a minor detail on a long problem list, but as one more way to care for the person behind it.

